# Autogenous fibula head transplantation for aneurysmal bone cyst of distal radius: A case report followed up for 7 years

**DOI:** 10.1097/MD.0000000000036210

**Published:** 2024-01-12

**Authors:** Zhi Wen, Gaoyan Kuang, Yong Jiang, Yuyuan Wu, Enxu Liu, Liguo Qiu, Xiaotong Xu, Min Lu

**Affiliations:** a Department of Joint Orthopedics, The First Hospital of Hunan University of Chinese Medicine, Changsha, Hunan, China; b Hunan University of Chinese Medicine, Changsha, Hunan, China; c Department of Pediatric Orthopedics, Traditional Chinese Medicine Hospital in Huaihua, Huaihua, Hunan, China.

**Keywords:** aneurysmal bone cyst (ABC), autogenous fibula transplantation, case report

## Abstract

**Rationale:**

Aneurysmal bone cyst (ABC) is a rare primary or secondary tumor that usually occurs in young women aged between 10 and 20 years, mostly in the long tubular bone and spine. However, there are no definite standards for its clinical treatment. To our knowledge, this is the first report of a young female patient with distal radius ABC who was successfully treated with tumor resection and autogenous fibular head transplantation.

**Patient concerns:**

A 28-year-old married Chinese young woman presented to our hospital with swelling and pain in her right wrist for 2 years and aggravation of wrist movement restriction for 1 week.

**Diagnoses:**

Pathological biopsy confirmed ABC.

**Interventions:**

We performed a pathological examination of the tumor on the right wrist and preliminarily confirmed the diagnosis of ABC. The right wrist joint was reconstructed by total surgical resection of the ABC tumor in the right wrist joint and autogenous fibular head transplantation.

**Outcomes:**

During follow-up within 7 years, good right wrist function was confirmed. The tumor did not recur, the swelling of the right wrist disappeared, the joint pain and limitation of movement significantly improved, and the function of the right wrist was not impaired in daily activities. Radiography showed that the fracture had healed.

**Lessons:**

Our results suggest that autofibular head transplantation is an effective treatment for reconstruction of wrist function in adult patients with ABC of the distal radius.

## 1. Introduction

Aneurysmal bone cyst (ABC) was first described by Jaffe and Lichtenstein in 1942.^[1]^ It is a benign, locally invasive tumor that occurs mostly in children and in early adulthood. It is common in the metaphyses of the long bones of the extremities and can also occur in the spine or pelvis.^[2,3]^ However, the exact etiology and pathogenesis of ABC remain unclear. The incidence of ABC is more than 75% in patients under 20 years of age, but it can also occur in larger patients,^[4,5]^ and the incidence rate of ABC is higher in women than that in male.^[6]^ The diagnosis of ABC must be confirmed through tissue biopsy and histopathological examination. Histopathology shows that the disease is composed of cysts of different sizes and connective tissue septa, and the cysts are full of blood.^[5]^

There is no definite standard for the treatment of ABC, and the best treatment remains under discussion.^[7]^ Different doctors describe different treatments, such as intratumoral curettage, embolization, local percutaneous drug injection, cryoablation, radiotherapy, and the systemic application of denosumab or bisphosphate.^[8,9]^ ABC grows rapidly and is destructive to local tissues; therefore, routine treatment is accompanied by possible complications and easily leads to recurrence. Block resection of the tumor may cause serious growth disorders and lead to large bone defects in children; therefore, minimally invasive surgery is preferred.^[10]^ However, all non-block resection treatments have a risk of local recurrence.^[11]^ In this case, the patient was a 28-year-old female patient with ABC. Because the tumor is large and has affected the movement of the joint, we estimate that the effect of conventional treatment is limited and can easily recur, so we drew up a plan for surgical resection of the tumor and wrist reconstruction at the same time. Reconstruction surgery is challenging, and it is difficult to select a suitable graft that can match the shape of the distal radius, because the fibula head is physiologically similar to the distal radius, and the fibula does not participate in the weight-bearing of the lower extremities and the activity of the knee joint. The autogenous bone grafts did not appear to be rejected.

## 2. Case report

**Figure 1.**
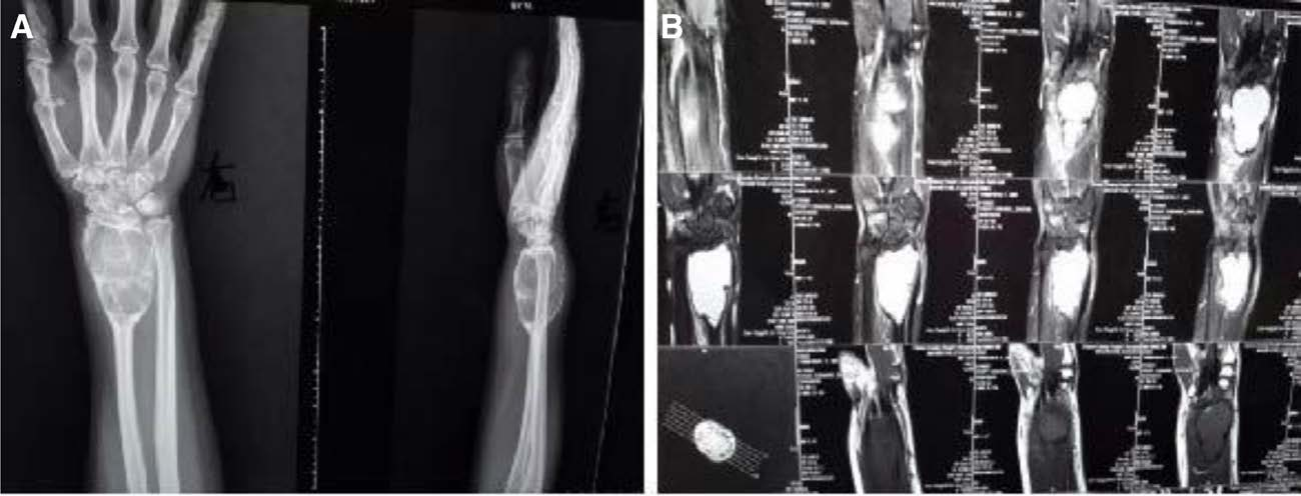
Imaging examination. (A) X-ray showed osteolytic and expansive destruction of the distal right radius tumor with clear boundary and multilocular changes formed by bony septum. (B) MRI showed cystic changes in the distal right radius tumor with clear boundary, low signal intensity on MRI T1WI and high signal intensity on MRI T2WI, and the tumor did not break through the articular surface of the distal radius. MRI = magnetic resonance imaging.

The patient was admitted to the hospital because of swelling and pain in the right wrist for 2 years and aggravation with limited joint movement for 1 week. More than 2 years ago, because of the tendon sheath cyst on the back of the right wrist, puncture aspiration and drug treatment of the tendon sheath cyst were performed in a local hospital (the specific drugs are unknown). Subsequently, faint pain began to appear in the right wrist. Later, owing to the birth and nursing of the baby, she did not continue to undergo examination and treatment, and the swelling and pain in her right wrist gradually worsened. One week before admission, X-ray and nuclear magnetic resonance imaging (MRI) were performed in the local county hospital, and a giant cell tumor of the distal right radius was considered (Fig. 1).

**Figure 2.**
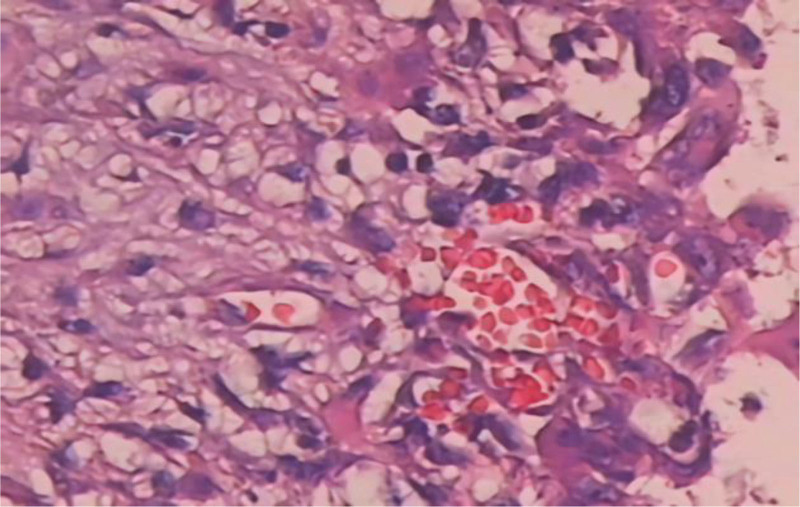
The first pathological examination. Diagnosed as ABC. ABC = aneurysmal bone cyst.

The relevant examination findings further improved after hospitalization in our hospital. *History of past illness*: only a history of small intrahepatic hemangiomas. *Personal and family history*: Personal and family histories, medication history, social history, and allergic history were negative. *Physical examination*: The right wrist joint showed obvious swelling, normal skin temperature, local tenderness, unpalpable bone friction and abnormal activity, limited flexion, extension, and rotation of the right wrist, and normal sensation and blood circulation of the right finger. *Laboratory examinations*: All laboratory tests were within the normal range. Routine blood tests (white blood cell count, red blood cell count, hemoglobin, etc), ESR, C-reactive protein, rheumatoid factor, female tumor marker levels, and urine and stool tests were also normal. First, we obtained a biopsy sample. Under nerve block anesthesia, samples were taken through a small skin incision on the right wrist. By cutting the bone surface of the tumor, a large amount of blood was found in the lesion. Part of the blood in the lesion was drawn, and a small amount of bone tissue was excised from the tumor wall for examination. A pathological biopsy confirmed ABC (Fig. 2).

**Figure 3.**
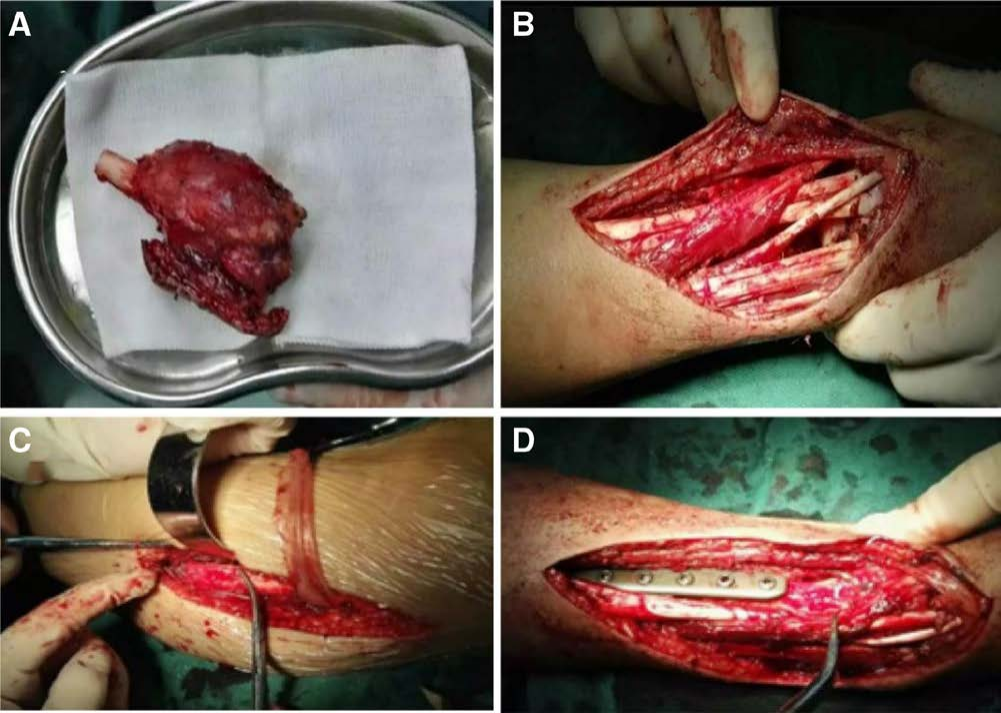
Operation: (A and B) under general anesthesia, the tumor was fully exposed through an S-shaped skin incision on the dorsal radial side of the right wrist, and the tumor was completely removed from the articular surface of the distal radius to the normal bone of the proximal radius, with a length of 8 cm. (C and D) At the same time, the operation was performed on the contralateral fibula. Protect the common peroneal nerve, cut off the length of the fibular head to 8 cm, and retain the capsule wall and tendon attachment on the fibular head. The autogenous fibular head was transplanted to the right wrist and fixed with 6-hole steel plate and 2 Kirschner wires.

**Figure 4.**
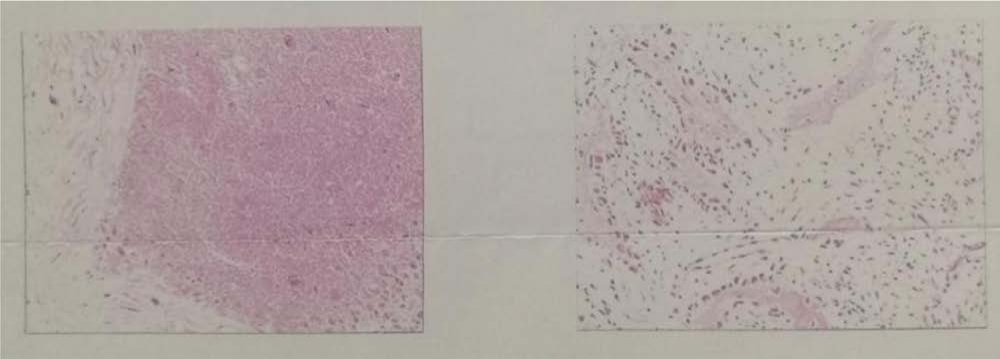
Second pathological examination: re-diagnosed as ABC. ABC = aneurysmal bone cyst.

**Figure 5.**
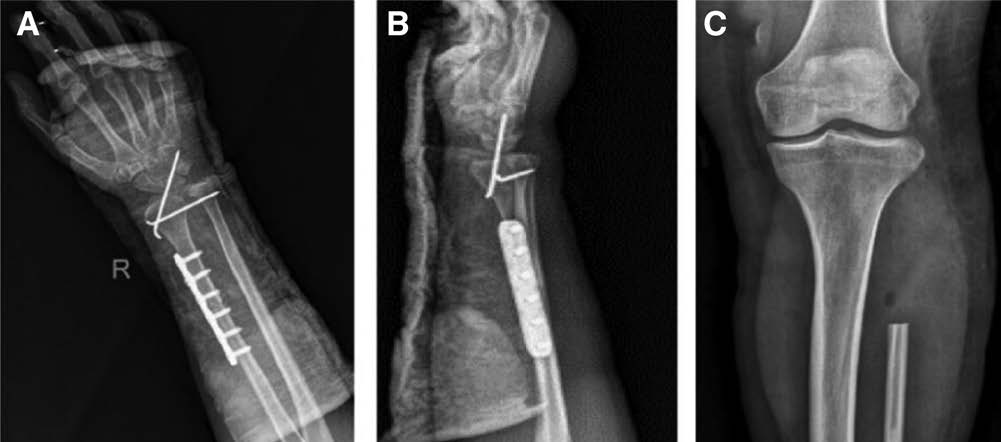
X-ray examination after operation. (A and B) The position of the fibular head transplanted at the wrist joint was good, and the shape of the reconstructed wrist joint was satisfactory. (C) X-ray findings of contralateral fibular head after removal.

**Figure 6.**
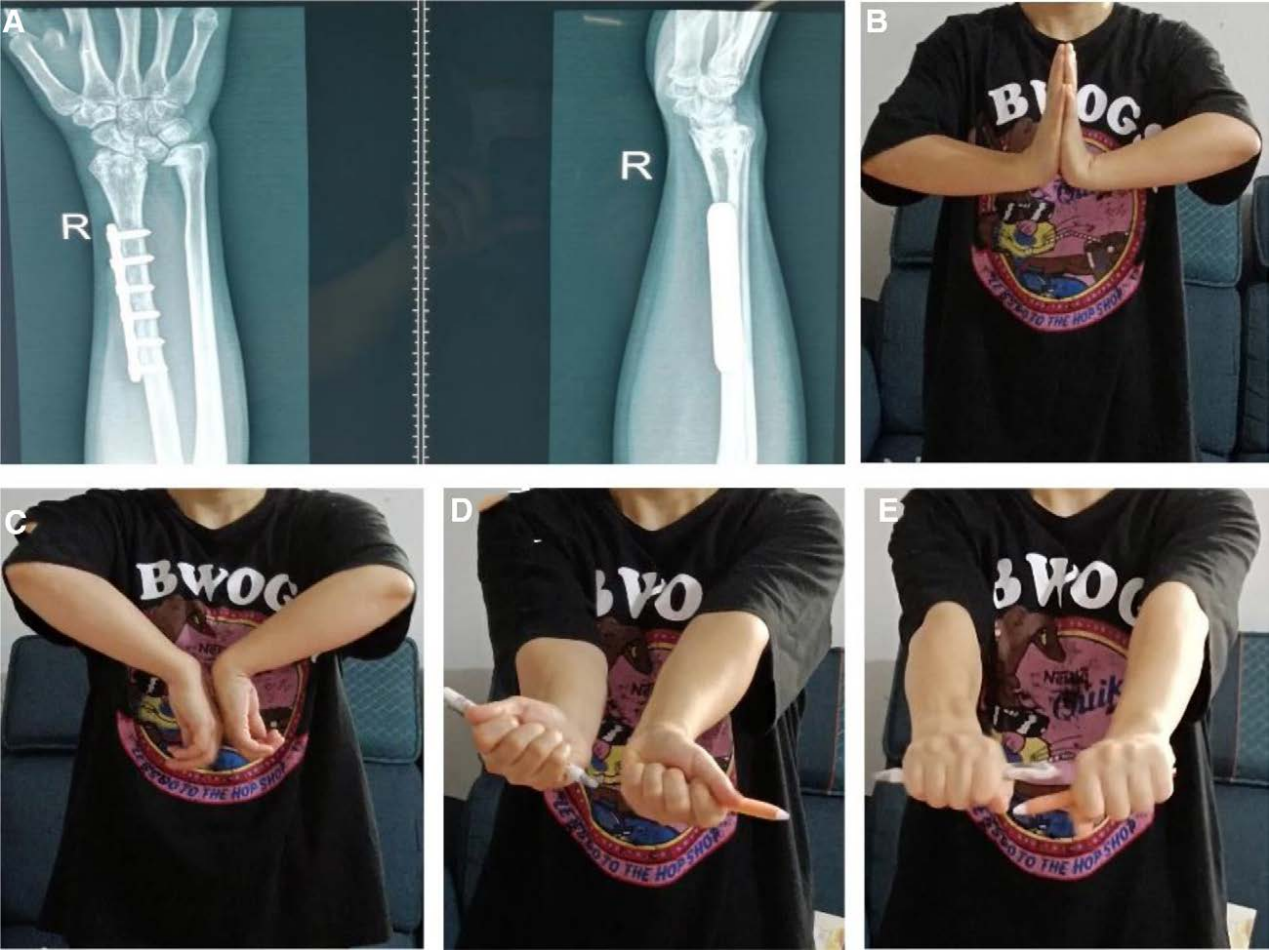
(A) Reexamination 2 yr after operation: the X-ray showed that the bone reconstruction had healed and the articular surface of the fibular head was fine. (B)The function of dorsal extension of wrist joint is close to normal. (C) The metacarpal flexion function of the wrist is about 30º worse than that of the contralateral side. (D) The external rotation function of the wrist is about 15º worse than that of the contralateral side. (E) The internal rotation function of the wrist is close to normal.

**Figure 7.**
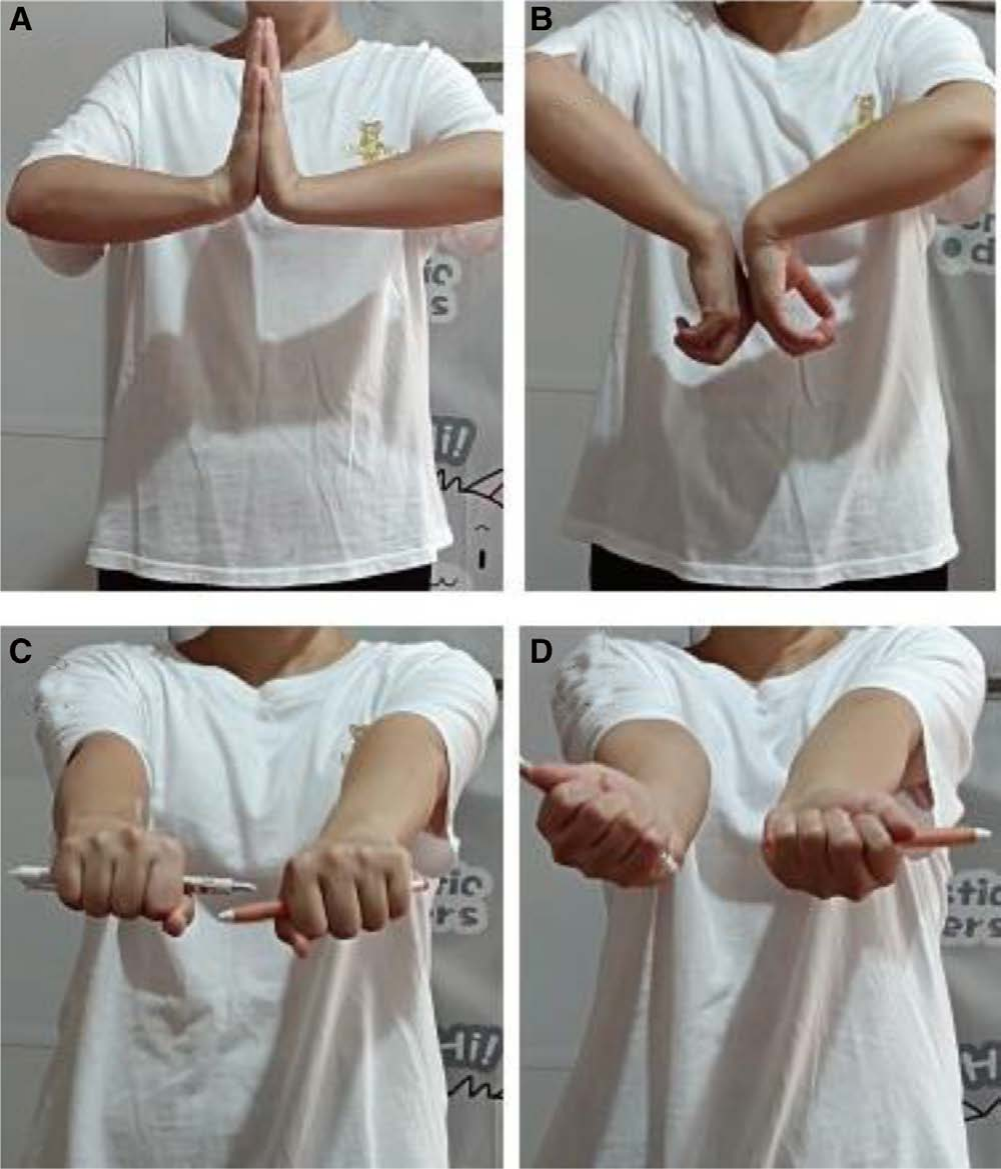
In the 7th yr after operation, the function of dorsal extension of wrist joint was normal, the function of metacarpal flexion was improved, the function of internal rotation was normal, and the difference of external rotation was about 10º compared with the healthy side.

Due to the large size of the tumor, the effect of conventional treatment of ABC is limited; therefore, we have developed a surgical plan for the reconstruction of wrist function by surgical resection of the tumor and autogenous fibular head transplantation. Under general anesthesia, the tumor was fully exposed through an S-shaped skin incision on the dorsal radial side of the right wrist, and was completely removed from the articular surface of the distal radius to the normal bone of the proximal 8 cm (Fig. 3A and B). At the same time, the surgery was performed on the contralateral fibula of the patient, the skin was cut open, the common peroneal nerve was exposed and protected, the fibula was cut off from the fibular head to the distal 8 cm, and the capsule wall and tendon attachment on the fibular head were preserved. The autogenous fibular head was transplanted into the right wrist and fixed with 6-hole steel plate and 2 Kirschner wires (Fig. 3C and D). After the operation, the wrist was fixed with plaster, and the tumor was again diagnosed as ABC (Fig. 4). Three days after surgery, radiographs of the right wrist and left knee joint (Fig. 5A–C) showed that the position of the fibular head transplanted at the wrist joint was good, and the shape of the reconstructed wrist joint was satisfactory. Three weeks after the operation, 2 Kirschner needles were removed from the right wrist joint under local anesthesia. *Outcome and follow-up:* After the operation, the tumor was completely removed, swelling of the right wrist disappeared, and joint pain and limitation of movement were significantly improved. Two years after the operation, the right forearm and wrist joint were in good shape; there was no pain, swelling, or obvious deformity; the range of flexion and extension of the wrist was good; the supination angle was 15º less than that of the contralateral side; and the pronation function of the right wrist was good. Radiography showed that the fracture had healed (Fig. 6A–E), and the plates and screws were removed under nerve block anesthesia 2 years after the operation. In the 7th year after the operation, the function of dorsal extension of the wrist joint was normal, the function of metacarpal flexion was improved, the function of internal rotation was normal, and the difference in external rotation was approximately 10º compared with the healthy side (Fig. 7A–D).

## 3. Discussion

ABC is a rare benign tumor; however, few clinical cases have been reported,^[12]^ and it is divided into primary ABC and secondary ABC. Primary ABC accounts for 70% of the total incidence. Secondary ABC is most common in fibrous dysplasia, non-ossifying fibroma, giant cell tumor of the bone, chondroblastoma, and so on. X-ray and MRI examinations are helpful to distinguish ABC from some diseases; patients are usually treated for swelling and pain in the lesion area, and sometimes pathological fractures can be observed. A relatively clear osteolytic and dilated lesion can be found on the X-ray film, and MRI shows a typical cystic structure in the liquid-liquid plane due to blood deposition.^[13]^ Tissue biopsy is necessary, and the results of pathological examination must be observed together with clinical symptoms, medical history, and imaging examination. ABC grows rapidly and is highly destructive to the surrounding tissues. The interior of the ABC can be divided into many hyperemia cavities of different sizes by thickening the diaphragm, and the fibrous septum is composed of osteoclast-like giant cells, matrix monocytes, spindle cells, and reactive braided bone.

Percutaneous alcohol sclerotherapy is effective for the treatment of ABC in children, but the size, invasiveness, location, and age of the patients should be considered.^[14]^ Li^[15]^ reported a 57-year-old patient with a giant ABC secondary to a giant cell tumor of the finger bone who was finally treated with amputation of the fifth finger. Denosumab seems to be effective in relieving pain and improving nerves in ABCs, but it does not significantly reduce the actual recurrence rate and has potentially life-threatening side effects.^[16,17]^ Crowe^[18]^ uses a high speed grinding head and autogenous bone graft to reduce the probability of ABC recurrence to some extent. In the conventional treatment, the recurrence rate of local resection combined with bone grafting and radiation resection is 36%, and that of curettage or curettage combined with bone grafting is 79%; therefore, extensive resection is considered to be the most effective way to prevent recurrence.^[19]^ Malignant transformation of ABC is rare, and there is a certain probability of malignant transformation if it recurs many times. Song^[20]^ reported a case of an ABC that transformed into an osteosarcoma. Accurate diagnosis of ABC is very important for treatment decision-making, and pathological examination is the standard for the diagnosis of ABC. Therefore, we performed a local pathological examination before choosing complete resection and then made a second-stage treatment plan after the diagnosis was confirmed. The treatment experience of previous studies is mainly aimed at young patients in the period of growth and development, and there are no specific cases of autogenous fibular head transplantation. Based on the patient condition, we used autogenous fibular head transplantation to reconstruct the wrist joint of the patient. The function of the wrist joint recovered satisfactorily and there was no recurrence. Therefore, this treatment scheme can be used as a reference for special cases.

ABC is easily misdiagnosed as a giant cell tumor of the bones. Accurate diagnosis of ABC is very important for treatment decision-making, and pathological examination is the standard for the diagnosis of ABC. We first performed pathological examination and preliminarily confirmed the diagnosis of ABC. The right wrist joint was reconstructed by total surgical resection of the right wrist ABC tumor and autogenous fibular transplantation. During the 7-year follow-up, good function of the right wrist and basic recovery of daily life were observed. To our knowledge, this is the first report of successful autogenous fibular head transplantation and satisfactory functional recovery in a young patient with distal radius ABC.

## Author contributions

**Data curation:** Zhi Wen, Min Lu.

**Investigation:** Zhi Wen, Yong Jiang, Yuyuan Wu, Enxu Liu.

**Methodology:** Liguo Qiu.

**Project administration:** Yong Jiang, Yuyuan Wu, Min Lu.

**Writing – original draft:** Zhi Wen, Enxu Liu.

**Writing – review & editing:** Gaoyan Kuang, Liguo Qiu, Xiaotong Xu.
